# Correlation between elastic energy stored in an eye and visual field progression in glaucoma

**DOI:** 10.1371/journal.pone.0204451

**Published:** 2018-09-21

**Authors:** Shuichiro Aoki, Hiroshi Murata, Shunsuke Nakakura, Yoshitaka Nakao, Masato Matsuura, Yoshiaki Kiuchi, Ryo Asaoka

**Affiliations:** 1 Department of Ophthalmology, The University of Tokyo, Tokyo, Japan; 2 Department of Ophthalmology, Sapporo City General Hospital, Sapporo, Japan; 3 Department of Ophthalmology, Saneikai Tsukazaki Hospital, Hyogo, Japan; 4 Department of Ophthalmology and Visual Science, Hiroshima University, Hiroshima, Japan; 5 Orthopic and Visual Science, Department of Rehabilitation, School of Allied Health Sciences, Kitasato University, Kanagawa, Japan; Faculty of Medicine, Cairo University, EGYPT

## Abstract

**Purpose:**

To investigate whether the elastic energy stored in an eyeball at highest concavity (highest concavity energy; HCE), calculated with Corivs ST (CST, OCULUS), correlates with glaucomatous visual field (VF) progression.

**Methods:**

108 eyes from 70 primary open angle-glaucoma patients were studied. The HCE was calculated using CST parameters. For each eye, the mean total deviation (mTD) of the 52 test points in the 24–2 Humphrey Field Analyzer test pattern was calculated and the mTD progression rate was determined from eight reliable VFs. Eyes were subdivided into: subgroups with low- or high-whole eye motion maximal length (WEM-d) and subgroups with short- or long-time taken to reach WEM-d (WEM-t), as measured with CST. The associations between mTD progression rate and HCE and other ocular/systemic parameters including age, Goldmann applanation tonometry based-intraocular pressure [GAT-IOP], and corneal hysteresis [CH] from the Ocular Response Analyzer (ORA^®^, Reichert) were investigated using the linear mixed model. The optimal model to describe mTD progression rate was selected from all possible combinations according to the second order bias corrected Akaike Information Criterion index.

**Results:**

Optimal models to describe mTD progression rate included: CH in the model for all eyes, age and HCE in the model for the WEM-d low group, HCE in the model for the WEM-t short group, mean GAT-IOP in the model for the WEM-d high group, and age in the model for the WEM-t long-group.

**Conclusions:**

HCE was associated with glaucomatous VF progression in eyes with minimal whole eye motion (low WEM-d and WEM-t subgroups).

## Introduction

Intraocular pressure (IOP) is the main risk factor for the development and progression of glaucoma.[1–5] However, previous studies have shown that lowering IOP does not completely suppress visual field progression in all patients,[6, 7] which suggests the importance of seeking other risk factors related to the development and progression of glaucoma. In particular, biomechanical properties of the eye are known to play a significant role in the development and progression of glaucoma. Many reports have suggested a close relationship between corneal hysteresis (CH) measured with the Ocular Response Analyzer (ORA, Reichert Ophthalmic Instruments, Depew, NY, USA)[8–10] and the development and progression of glaucomatous visual field (VF) defects.[11–14] Elucidating this clinical relationship between CH and chronic glaucomatous damage appears challenging, considering the fact that CH is merely the pressure difference at two time points within a very short measurement time within a few hundred microseconds and that it still remains somewhat debatable as to what it really represents. Some researchers explain this relationship in terms of similarity in tissue constitutions; the cornea, lamina cribrosa and peripapillary sclera have biomechanical properties in common[15–18], and consequently, a higher CH may somehow represent a more energy-dissipative structure at the optic nerve head. Considering recent studies that revealed eyes receive daily incessant stress in the form of ocular microtremors[19], pulsatility[20], blinking,[21, 22] and general eye movements,[23, 24] we hypothesize in the current study that external stress that occurs repeatedly within a short period of time contribute to glaucomatous change by giving excessive energy load to the optic disc. When external stresses are placed on an eyeball, some energy is absorbed by the damping capacity of an eye, but the remaining unabsorbed energy can be a stress on the posterior segments of an eye, including the structures around the optic nerve head, which can cause damage to the axons and eventually the retinal ganglion cells.

Corvis ST (OCULUS: CST, Ver 1.13b1361) is a relatively new device designed to record the response of the cornea after application of an air-pulse pressure.[25, 26] The instrument captures a sequence of images with high temporal resolution and various parameters are derived, including corneal deformation amplitude, length, area and time. Currently, CST does not provide any measure for biomechanical property of an eye. However, this study proposes that it is possible to assess the magnitude of stored elastic energy at the highest concavity (HC) state (HCE). This is an important measurement because HCE plus the magnitude of energy dissipation (damping) must be approximately equal to the amount of applied energy, which is a constant value in the CST measurement. This means that HCE serves as a surrogate measure for the magnitude of energy dissipation, because the smaller the former is, the larger the latter is. This inverse correlation becomes weak if the magnitude of energy consumption due to friction between the eye ball and orbital tissue, caused by whole eye motion (WEM), is large.

In our previous studies[27–29], the usefulness of raw parameters of CST on the severity and progression of glaucoma was evaluated and it was speculated that their relations are because eyes with a rich energy dissipation are advantageous to avoid rapid progression of glaucoma. However, it has not been elucidated yet. Thus, in this study, we developed a method to approximate HCE, using CST parameters. We then show that this calculated energy measurement is closely related with the progression of glaucomatous VF damage, when WEM is minimal.

## Method

This retrospective study was approved by the Research Ethics Committee of the Graduated School of Medicine and Faculty of Medicine at The University of Tokyo. Written informed consent was given by patients for their information to be stored in the hospital database and used for research. This study was performed according to the tenets of the Declaration of Helsinki.

### Subjects

108 eyes of 70 primary open angle-glaucoma patients (37 males and 33 females) were included in this study. All patients had at least eight reliable VFs measured with the Humphrey Field Analyzer II (HFA, Carl Zeiss Meditec Inc, Dublin, CA), with the 24–2 or 30–2 and SITA standard program. VFs were deemed reliable if fixation loss (FL) rate <20% and false positive (FP) rate <15%; false negative (FN) rate was not used as an exclusion criterion, following the recommendation of the manufacturer. All patients had experience of VF testing prior to observation in the current study. We chose a minimum of eight VFs because it has been reported that this number is needed to precisely analyze VF progression.[30–34] Inclusion criteria were no abnormal eye-related findings except for primary open angle glaucoma on biomicroscopy, gonioscopy and funduscopy. Eyes with a history of other ocular disease, such as age-related macular degeneration, or eyes that had undergone surgery, including cataract and trabeculectomy surgery, during or prior to this VF series period were excluded. Only subjects aged ≧20 years old were included and contact lens wearers were excluded. The mean and standard deviation (SD) of all Goldmann applanation tonometry intraocular pressure (GAT-IOP) measurements during the follow up period were calculated. Corneal curvature, axial length (AL) and central corneal thickness (CCT) were also measured in all patients using TONOREF II (NIDEK), IOL Master ver. 5.02 (Carl Zeiss Meditec, CA) and CST, respectively.

### VF data

The mean total deviation (mTD) value of the 52 test points in the 24–2 HFA VF was calculated. The progression rate of mTD was determined using the eight VFs collected from each eye, similar to the MD trend analysis employed in the HFA Guided Progression Analysis (GPA).

### ORA measurements

ORA records two applanation pressure measurements, prior to and following indentation of the cornea with the application of a rapid air jet. Due to its viscoelastic property, the cornea resists the air puff, resulting in delays in the inward and outward applanation events, which causes a measurable difference in the air puff values. This difference is called CH. ORA also provides corneal compensated IOP (IOPcc), which is an IOP measurement adjusted for corneal properties.[35] ORA measurements were carried out three times with at least a five minute interval between each measurement, prior to and on the same day of the GAT-IOP measurement within 180 days from the eighth VF measurement. The order of ORA and CST measurements was decided randomly. Only data with a quality index > 7.5 were analyzed. The average values of CH and IOPcc from the three measurements were used for analysis; corneal resistance factor (CRF) was not used since previous studies have indicated CH, but not CRF, is associated with glaucomatous progression.[12–14, 36]

### Corvis ST measurement

**Fig 1:**
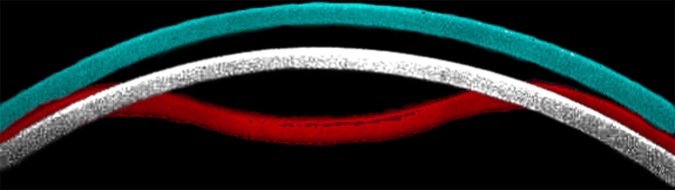
An illustration of corneal apical movement and ‘Whole Eye Motion’ in the CST measurement. Corneal locations at Whole Eye Motion (white, at 22.41 ms from the initiation of air jet) and maximum deformation concavity (red, at 16.40 ms from the initiation of air jet)) are superimposed on the cornea prior to the CST measurement (blue). In this case, the time of the second applanation (‘A2 deformation time’) was 22.05 ms. CST: CorvisST.

The principles of CST have been described in detail elsewhere.[37] In short, the instrument’s ultra-high speed camera records 140 images of corneal deformation for 30 ms from the initiation of an air puff application. During the inward and outward movements, the cornea experiences two applanations, between which the HC moment takes place. The magnitude and the timing of the air puff is constant across all measurements. CST also measures the backward movement of the peripheral cornea as WEM (**Fig 1**), which is summarized by two parameters: the maximal displacement of WEM (WEM-d) and the time to reach the WEM state (WEM-t). Additionally, IOP is measured and corrected according to age, CCT and HC radius; this measurement is known as biomechanically corrected IOP (bIOP).[38]

CST (software version; ver. 1.13b1361) measurements were performed three times on the same day as the ORA measurements, with at least a five-minute interval between each measurement. Only reliable CST measurements, according to the “OK” quality index displayed on the device monitor, were used. The average values of CCT, WEM-d, WEM-t, peak distance at HC, corneal radius at HC were used for the calculation of HC elastic energy (HCE).

### Calculation of the stored elastic energy at the HC time (HCE)

The HCE was calculated as the amount of energy required for the corneal movement from the HC state to the recovered (un-deformed) state. This can be explained using a simple mechanical model of a syringe (representing the eye’s anterior chamber) and a rubber balloon (representing the eye’s vitreous chamber); the open end of the syringe is connected to the balloon and both are filled with fluid (**S1 Fig**). When the syringe is pressed to insert fluid into the balloon, the change in volume in the syringe is identical to the change in volume in the balloon, because the volume change of the fluid is negligible. The inflated balloon stores an elastic energy and this energy contracts the balloon resulting in movement of fluid into the syringe, when the pressure to the syringe is released. The amount of this kinetic energy is identical to that of the stored elastic energy in the balloon, assuming no elastic properties of the fluid and syringe barrel. In the case of an eye, the syringe and balloon correspond to the anterior chamber and vitreous chamber, respectively; the amount of elastic energy stored in the stretched sclera at the HC time can be calculated by integrating the cornea displacement with IOP. It should be noted that there is no kinetic energy at the corneal apex at the HC state, because the corneal apex is paused. Also, the energy translations are different before and after the HC state; after the point of HC, elastic energy no longer augments but dissipates.

The details of the HCE calculation are shown in **S2 Fig**. In short, HCE was calculated using the corneal movement volume between the original shape and the HC state (approximated using the CST derived peak distance and corneal radius at HC), corneal curvature (from the keratometry measurement), and finally IOP (ORA-derived IOPcc). We assumed that the IOP value was constant throughout the corneal inward movement.

### Statistical analysis

The relationship between HCE and other ocular/systemic parameters (age, gender, CCT, AL, initial mTD, CH) was investigated using the linear mixed model, whereby a patient was registered as a random effect (because one or two eyes of a patient were included in the current study). Then, the association between mTD progression rate and the eight ocular/systemic parameters (age, mean GAT-IOP, SD of GAT-IOP, CCT, AL, mTD in the initial VF, CH, HCE) was also investigated using a linear mixed model. The optimal linear mixed model to describe the mTD progression rate was identified according to the second order bias corrected Akaike Information Criterion (AICc) index from all possible 2^8^ permutations. The AICc is a corrected form of the common AIC measurement, giving an accurate estimation even when the sample size is small.[39] Any magnitude of reduction in AICc suggests an improvement of the model,[40, 41] however, the relative likelihood that one particular model minimizes information loss is calculated as exp((AICc_min_ − AICc_x_)/2), where AICc_x_ is AICc value of arbitrary model “X” and AICc_min_ is the minimum AICc value from all possible models.[42] Correction for multiple testing was performed using Bonferroni’s method.[43]

In order to take account of WEM on the relationship between HCE and glaucomatous VF progression rate, the average values of WEM-d and WEM-t were calculated and then eyes were divided into subpopulations. Eyes with WEM-d lower than the average value were classified as WEM-d low-group and eyes with WEM-d higher than the average value as WEM-d high-group. Similarly, eyes with WEM-t shorter than the average value were classified as WEM-t short-group and eyes with WEM-t longer than the average value as WEM-t long-group. The association between mTD progression rate and the eight ocular/systemic parameters (age, mean GAT-IOP, SD of GAT-IOP, CCT, AL, mTD in the initial VF, CH, HCE) was investigated using a linear mixed model in all eyes as well as each subgroup. Marginal R-squared value for a linear mixed model was calculated following a method proposed by Nakagawa and Holger.[44]

All statistical analyses were performed using the statistical programming language ‘R’ (R version 3.2.3; the foundation for Statistical Computing, Vienna, Austria)

## Results

Characteristics of the subjects, as well as CH and HCE values, are summarized in **Table 1**. The mean ± standard deviation (SD) [range] age was 53.9±10.2 [32 to 79], and eight VFs were measured over the period of 2257.3±983.6 [371 to 6895] days. GAT-IOP was conducted 29.0±7.1 [18 to 69] times during this period and mean GAT-IOP during the observation period was 13.5±2.2 [8.9 to 20.2] mmHg. As shown in the **Table 2**, mTD progression rate was -0.25±0.32 [-1.78 to 0.27] dB/year. There was no significant difference in the mTD progression rates between WEM-d low and high groups and also between WEM-t short and long groups (p = 0.44 and 0.48, respectively, linear mixed model).

**Table 1:** Summary of basic demographics.

Variables	Value
age, (mean ± SD) [range], years old	53.91 ± 10.23 [32.68 to 79.00]
Male / Female	37 / 33
Right / Left	56/ 52
GAT-IOP, (mean ± SD) [range], mmHg	13.47 ± 2.26 [8.93 to 20.19]
AL, (mean ± SD) [range], mm	25.13 ± 1.64 [22.30 to 29.20]
CCT, (mean ± SD) [range], μm	530.9 ± 35.82 [458.3 to 624.3]
Initial mTD, (mean ± SD) [range], dB	-5.72 ± 5.44 [-22.44 to 2.48]
CH, (mean ± SD) [range], mmHg	9.16 ± 1.14 [6.50 to 11.79]
HCE, (mean ± SD) [range], mmHg*mm^3^	42.82 ± 8.65 [14.66 to 76.30]

AL: axial length, CCT: central corneal thickness, CH: corneal hysteresis, CST: CorvisST, GAT-IOP: Goldmann applanation tonometry-based intraocular pressure, HCE: elastic energy at highest concavity, mTD: mean of total deviations, SD: standard deviation

**Table 2:** mTD progression rate in all eyes, WEM-d low, WEM-d high, WEM-t short, and WEM-t long subgroups.

Group	mTD progression rate (coefficient±SE, [range))	P value
All	-0.25±0.32 [-1.78 to 0.27]	-
WEM-d low group	-0.24±0.36 [-1.78 to 0.27]	0.44
WEM-d high group	-0.26±0.27 [-0.91 to 0.23]	
WEM-t low group	-0.26±0.35 [-1.78 to 0.27]	0.48
WEM-t high group	-0.24±0.29 [-1.26 to 0.21]	

mTD: mean of total deviations, SE: standard error, WEM: whole eye motion

The univariate relationship between HCE and the values of age, gender, CCT, AL, GAT-IOP on the same measurement day, CH, WEM-d and WEM-t are shown in the **Table 3**; significant relationships were observed between HCE and CCT, CH, AL, GAT-IOP on the measurement day and WEM-d.

**Table 3:** The relationship between elastic energy parameters and age, CCT, CH, AL, GAT-IOP on the measurement day, WEM-d and WEM-t.

	HCE		
	Coefficient	SE	p value
Age	-0.1610	0.094	0.10
CCT	-0.072	0.026	0.0096
CH	-3.99	0.68	<0.001
AL	2.90	0.49	<0.001
GAT-IOP on the measurement day	-1.31	0.41	0.0028
WEM-d	-37	12	0.0031
WEM-t	0.033	1.120	0.98

AL: axial length, CCT: central corneal thickness, CH: corneal hysteresis, GAT-IOP: Goldmann applanation tonometry-based intraocular pressure, HCE: elastic energy at highest concavity, SE: standard error, WEM: whole eye motion

The optimal model for HCE was: HCE = 7.11–4.12 (standard error: SE = 0.50, p < 0.001) x CH + 2.93 (SE = 0.35, p < 0.001) x AL.

The univariate relationship between mTD progression rate and the values of age, gender, CH, CCT, AL, mean GAT-IOP, WEM-d, WEM-t and HCE in all eyes are shown in **Table 4**. Mean GAT-IOP was not significantly related to mTD progression rate in all eyes or any subgroup (WEM-d low, WEM-d high, WEM-t short and WEM-t long groups; p = 0.43, 0.27, 0.52, 0.12 and 0.54, respectively). CH was significantly related to mTD progression rate in all eyes and the WEM-d low group (p = 0.011 and 0.034, respectively), but not in the WEM-d high, WEM-t short and WEM-t long groups (p = 0.22, 0.089 and 0.085, respectively). HCE was significantly related to mTD progression rate in the WEM-d low group and WEM-t short groups (p = 0.040 and 0.012, respectively), but not in all eyes, the WEM-d high group and WEM-t long group (p = 0.082, 0.84 and 0.62, respectively).

**Table 4:** The relationship between the values of mTD progression rate and age, gender, CCT, CH, AL, mean GAT-IOP, and HCE, with p values from the linear mixed model.

	mTD progression rate			
	Coefficient	SE	p value	AICc
Age	-0.0049	0.0029	0.10	64.66
Gender	0.0057	0.060	0.93	67.39
CCT	0.0014	0.00084	0.092	64.49
CH	0.068	0.026	0.011	60.88
AL	0.0039	0.019	0.83	67.35
Mean GAT-IOP	0.011	0.014	0.43	66.76
HCE	-0.0065	0.0037	0.082	59.66
WEM-d	0.36	0.46	0.44	62.28
WEM-t	0.030	0.042	0.48	62.34

AICc: corrected Akaike Information Criterion, AL: axial length, CCT: central corneal thickness, CH: corneal hysteresis, GAT-IOP: Goldmann applanation tonometry-based intraocular pressure, HCE: elastic energy at highest concavity, mTD: mean of total deviations, SE: standard error, WEM: whole eye motion

As shown in **Table 5**, across all eyes, the optimal model for mTD progression rate was: mTD progression rate = -0.95 + 0.075 x CH (AICc = 55.79).

**Table 5:** The optimal models to describe mTD progression rate with explanatory variables selected from age, mean GAT-IOP, SD of GAT-IOP, CCT, AL, mTD in the initial VF, CH, and HCE.

Group	Variables	coefficient	SE		p value	AICc of optimal model	AICc of monovariate model
All eyes (108 eyes)	CH	0.075	0.028		0.011	55.79	55.79
	age	N.S.					61.10
	mean GAT-IOP	N.S.					61.35
	SD of GAT-IOP	N.S.					62.84
	CCT	N.S.					60.14
	AL	N.S.					62.84
	mTD in the initial VF	N.S.					61.23
	HCE	N.S.					59.66
WEM-d low-group (55 eyes)	age	-0.011	0.059	0.080		41.93	45.95
	HCE	-0.014	0.0053	0.023			43.10
	mean GAT-IOP	N.S.					47.94
	SD of GAT-IOP	N.S.					48.02
	CCT	N.S.					46.15
	AL	N.S.					48.03
	CH	N.S.					47.11
	mTD in the initial VF	N.S.					42.78
WEM-d high-group (53 eyes)	mean GAT-IOP	0.028	0.017	p = 0.12		17.66	17.66
	age	N.S.					20.07
	SD of GAT-IOP	N.S.					20.29
	CCT	N.S.					19.76
	AL	N.S.					20.27
	mTD in the initial VF	N.S.					19.93
	CH	N.S.					18.70
	HCE	N.S.					20.38
WEM-t short-group (54 eyes)	HCE	-0.015	0.0050	0.012		35.33	35.33
	age	N.S.					43.82
	mean of GAT-IOP	N.S.					41.02
	SD of GAT-IOP	N.S.					43.65
	CCT	N.S.					43.09
	AL	N.S.					41.81
	mTD in the initial VF	N.S.					43.17
	CH	N.S.					40.38
WEM-t long-group (54 eyes)	age	-0.012	0.0038	0.0077		18.10	18.10
	mean of GAT-IOP	N.S.					27.28
	SD of GAT-IOP	N.S.					27.11
	CCT	N.S.					24.99
	AL	N.S.					23.61
	mTD in the initial VF	N.S.					25.98
	CH	N.S.					23.9
	HCE	N.S.					27.05

AICc: corrected Akaike Information Criterion, mR^2^: marginal R squared value, AL: axial length, CCT: central corneal thickness, CH: corneal hysteresis, GAT-IOP: Goldmann applanation tonometry-based intraocular pressure, HCE: elastic energy at highest concavity, mTD: mean of total deviations, SE: standard error, VF: visual field, WEM: whole eye motion, N.S.: not selected

In the WEM-d low-group, the optimal model for mTD progression rate was: mTD progression rate = 0.96–0.011 x age– 0.014 x HCE (AICc = 41.93). The relative likelihood that this model was the optimal model compared to a model with CH only was 1.53. In the WEM-d high-group, the optimal model for mTD progression rate was: mTD progression rate = - 0.64 + 0.028 x mean GAT-IOP (AICc = 17.66). Marginal R-squared value for the optimal model was 16%. This decreased to 5% when only age is considered.

In the WEM-t short-group, the optimal model for mTD progression rate was: mTD progression rate = 0.37–0.015 x HCE (AICc = 35.33). The relative likelihood that this model was the optimal model compared to a model with CH only model was 12.50. In the WEM-t long-group, the optimal model for mTD progression rate was: mTD progression rate = 0.39–0.012 x age (AICc = 18.10). Marginal R-squared value for the optimal model was 15%.

## Discussion

In the current study, CST measurements were carried out in 108 eyes with primary open angle-glaucoma. Using CST parameters, the stored elastic energy in an eyeball at the point of HC was calculated and its relationship to mTD progression rate was examined. The mTD progression rate was associated with HCE in the WEM-d low group and the WEM-t short group, but not in all eyes, the WEM-d high group, or the WEM-t long group.

Mean mTD progression rate across all eyes was -0.25 dB/year. This is comparable to, or slightly slower than, previous reports derived from real world clinics, including those in Heijl et al.[45], De Moraes et al. [46], Araie et al.[47] and the Japanese multicentral database[7] (mean mTD progression rate of -0.80, -0.45, -0.25 and -0.26 dB/year, respectively). The mean GAT-IOP during the follow up period was 13.47 mmHg, which is also comparable to the same previous reports (between 18.1 and 20.2 mm Hg, mean IOP of 15.2, 10.3 and 13.5 mmHg, respectively). Mean GAT-IOP during the observation period was not significantly correlated with VF progression. However, this does not deny the importance of IOP, an established risk factor for the development and progression of glaucoma,[1–5] because the current data were derived from a real-world clinic with patients under hypotensive therapy (as supported by a mean GAT-IOP of 13.47 mmHg). Nonetheless, the current results highlight that efforts to identify new risk factors for the progression of glaucoma remain important. For example, CNTGS revealed glaucoma progression in 20% of normal tension glaucoma patients despite a 30% reduction in IOP in the 3 years following treatment initiation.[6]

ORA-measured CH is merely a measurement of the difference of pressures at the inward and outward applanations and not identical to the true hysteresis of an eye. The hysteresis of a viscoelastic material is calculated as the area within the closed stress-strain curve plotted during an application of a cyclic load. Some studies have attempted to measure the actual hysteresis or energy dissipation of the cornea. Ishii et.al.[48] calculated the “elastic hysteresis”, using a noncontact tonometer and a high-speed camera. In their method, however, the camera did not provide the precise shape of the cornea, including the corneal apex position, i.e., strain. Vellara et al.[49] plotted corneal apex position against air pulse pressure using CST and calculated the area within the loading and unloading curves (named as ‘corneal energy dissipation’), although the energy dissipation was calculated using only the position of the corneal apex. The current method differs from these previous approaches in that we focused on HCE, a surrogate measure for the energy dissipation (damping capacity) of the whole eye at the point of HC, calculated not from the position of the corneal apex, but using the position and the shape of cornea. As a result, it was suggested that HCE was significantly associated with glaucomatous VF progression in the groups with minimal WEM (WEM-d low and WEM-t short groups).

In agreement with previous reports[13, 14, 50], CH was significantly related to mTD progression rate in all eyes. However, this relationship was not observed in the subgroups with minimal WEM (WEM-d low and WEM-t short groups), and instead HCE was closely associated with mTD progression rate. HCE, as described above, approximates the elastic energy, stored mostly in the sclera, at highest concavity. The negative correlation with VF progression indicates that the more elastic energy that is stored, or the less energy that is dissipated, the more rapidly the VF deteriorates. This result is consistent with the finding that HCE was negatively correlated with CH in all eyes. Meanwhile, HCE had no significant association with mTD progression rate in the subgroups with greater WEM (WEM-d high and WEM-t long groups). A natural hypothesis is that a large amount of energy was expended (distance and duration) due to friction with the orbit in eyes with larger WEM-d and WEM-t values, leading to the weak association between HCE and dissipated energy at highest concavity.

We assumed a constant level of IOP (IOPcc) in the HCE calculation. However, IOP will be higher when the eye is deformed following the application of an air pulse. Thus, HCE will be deviated from the actual elastic energy by the increase in IOP. Although HCE was also calculated using other bIOP and GAT-IOP, the derived HCE values were not significantly associated with mTD progression rate in any group of eyes. This may be because the magnitude of elevation of IOP when an eye is deformed in response to air pulse application may be related to the visco-elasticity of the cornea and sclera, considering IOPcc is designed to compensate for corneal properties[35] and is highly correlated with CH.[51, 52] bIOP, not only adjusted for CCT and age, but also for cornea curvature at HC (personal communication with Oculus) may represent some aspects of corneal visco-elasticity, though the HCE measurement calculated using bIOP was not significantly related to mTD progression rate.

Previous reports suggested that a thin cornea is a risk factor for VF progression.[53, 54] The CCT values in the current study were comparable to those observed in a previous study,[53] where average value was 553.1 μm. however, we found no significant relationship between CCT and the mTD progression rate, in contrast to HCE. Agreeing with this, Medeiros et al. have suggested that CH is a more useful parameter for assessing glaucomatous VF progression than CCT.[14] Evidence from the United Kingdom Glaucoma Treatment Study suggests a similar finding.[55]

A notable limitation of the current study is that eyes with different types of glaucoma, which may have different biomechanical properties, were not studied. Indeed, it has been reported that CH in eyes with exfoliation glaucoma, primary angle closure glaucoma and pigmentary glaucoma is different from CH in eyes with primary open angle glaucoma.[56, 57] A further study should be carried out to shed light on this issue.

It should be also noted that the WEM-d and WEM-t, measured by the motion of the peripheral cornea, is not identical to the actual movement of the entire eyeball. The measurement could be affected by many other variables like extraocular muscles thickness or orbital volume. The current result should be further validated once a better method to more accurately measure the movement of the back part of an eyeball is established.

## Conclusion

We have developed a method to calculate the elastic energy stored in an eye ball at the highest concavity, using Corvis ST measurements, and investigated its relation to glaucomatous visual field progression. Our results suggest that this novel parameter is a useful measure to assess VF progression in eyes when whole eye motion is small.

## Supporting information

S1 Fig A schema of an eyeball’s response to a mechanical stress using a simple syringe and rubber balloon model. The syringe and balloon are filled with fluid inside. When the syringe is pushed, the balloon inflates and an elastic energy is stored in the balloon wall. When the syringe is released, the balloon contracts using the elastic energy stored in the balloon, and the syringe is pushed back. The total amount of elastic energy stored in the balloon is equal to the work performed in pushing the syringe piston through the fluid. The syringe and the balloon wall represent the anterior chamber and sclera of the eye, respectively. Thus, the amount of elastic energy stored in the stretched sclera at the point of highest concavity can be calculated by integrating cornea displacement with intraocular pressure. HC: highest concavity, IOP: intraocular pressure.
(TIF)

S2 Fig Schematic illustration of the calculation of elastic energy stored at highest concavity (HCE). Assuming a constant intraocular pressure (IOP) level throughout the corneal deformation process, HCE is calculated assuming a constant IOP value and anterior chamber-volume change due to the corneal deformation (V). V is made up of two spherical segments with a common base; A_1_: upper segment with radius of corneal curvature (K) and A_2_: the lower segment with radius of concaved curvature at highest concavity (R). Heights of these segments (h_1_ and h_2_) are calculated with peak distance (PD), the distances between the two highest points of the cornea at highest concavity, using Pythagorean theorem. Then, holds, where is circular constant. Here we define PD: peak distance.
(TIF)
