# Supplementary figures and images for: Correlation between elastic energy stored in an eye and visual field progression in glaucoma
Source: PLoS One. 2018 Sep 21;13(9):e0204451. doi: 10.1371/journal.pone.0204451 (PMC6150541; doi:10.1371/journal.pone.0204451)

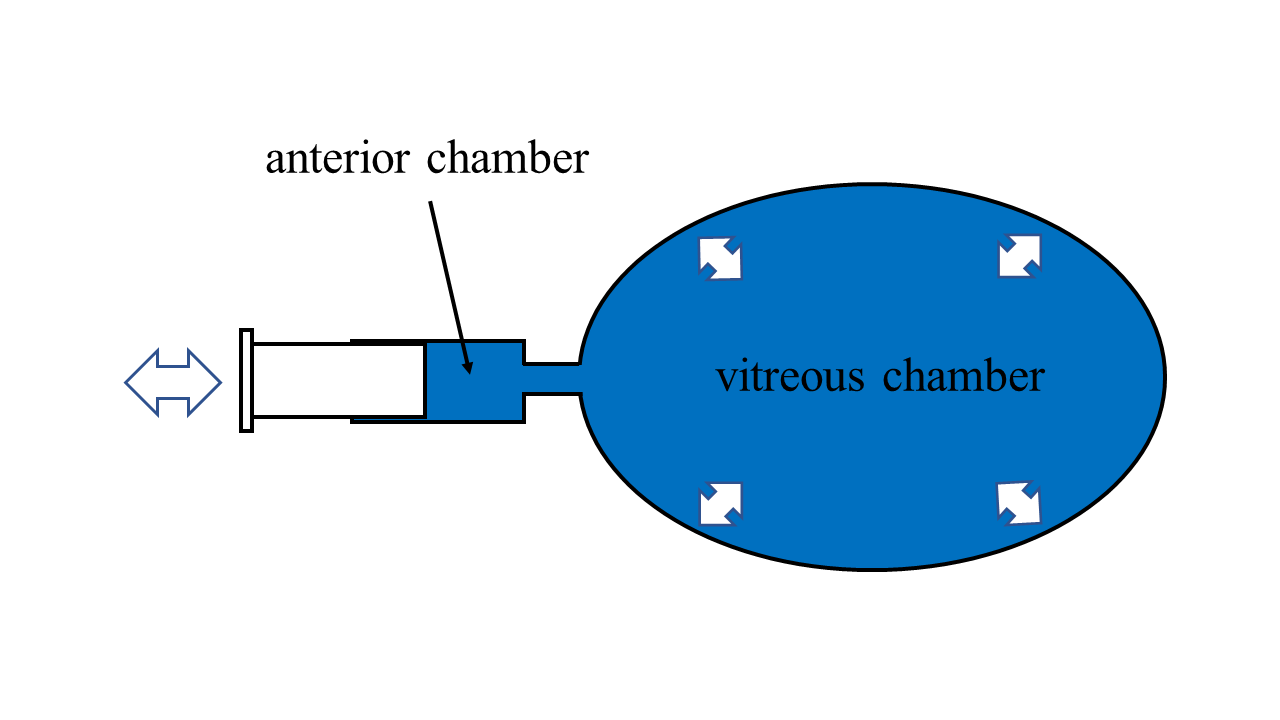

Supplement: S1 Fig — The syringe and balloon are filled with fluid inside. When the syringe is pushed, the balloon inflates and an elastic energy is stored in the balloon wall. When the syringe is released, the balloon contracts using the elastic energy stored in the balloon, and the syringe is pushed back. The total amount of elastic energy stored in the balloon is equal to the work performed in pushing the syringe piston through the fluid. The syringe and the balloon wall represent the anterior chamber and sclera of the eye, respectively. Thus, the amount of elastic energy stored in the stretched sclera at the point of highest concavity can be calculated by integrating cornea displacement with intraocular pressure. HC: highest concavity, IOP: intraocular pressure. (TIF) [file pone.0204451.s001.tif]

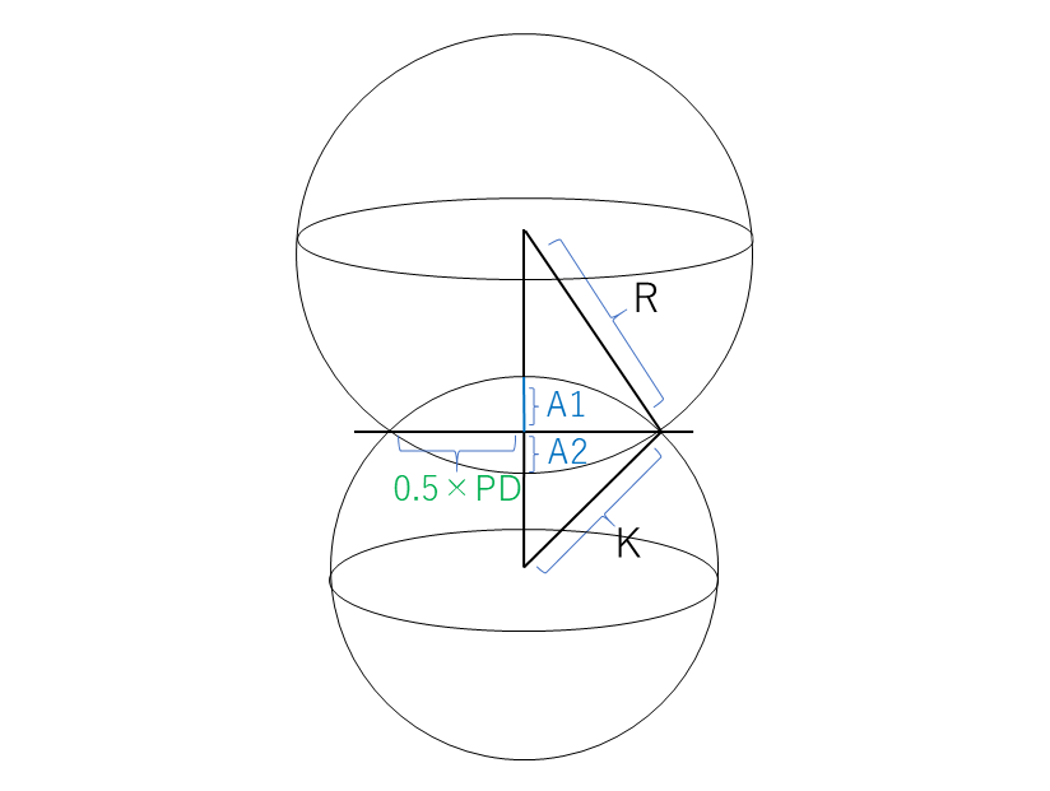

Supplement: S2 Fig — Assuming a constant intraocular pressure (IOP) level throughout the corneal deformation process, HCE is calculated assuming a constant IOP value and anterior chamber-volume change due to the corneal deformation (V). V is made up of two spherical segments with a common base; A1: upper segment with radius of corneal curvature (K) and A2: the lower segment with radius of concaved curvature at highest concavity (R). Heights of these segments (h1 and h2) are calculated with peak distance (PD), the distances between the two highest points of the cornea at highest concavity, using Pythagorean theorem. Then, holds, where is circular constant. Here we define PD: peak distance. (TIF) [file pone.0204451.s002.tif]
